# Effects of electroencephalography and regional cerebral oxygen saturation monitoring on perioperative neurocognitive disorders: a systematic review and meta-analysis

**DOI:** 10.1186/s12871-020-01163-y

**Published:** 2020-09-30

**Authors:** Lin Ding, Dong Xu Chen, Qian Li

**Affiliations:** National Clinical Research Center for Geriatrics and department of Anesthesiology, West China Hospital of Sichuan University & The Research Units of West China (2018RU012), Chinese Academy of Medical Sciences, Chengdu, 610041 China

**Keywords:** Electroencephalography, Regional cerebral oxygen saturation, Perioperative neurocognitive disorders, Postoperative delirium, Postoperative cognitive decline

## Abstract

**Background:**

Perioperative neurocognitive disorders (PND) is a common postoperative complication including postoperative delirium (POD), postoperative cognitive decline (POCD) or delayed neurocognitive recovery. It is still controversial whether the use of intraoperative cerebral function monitoring can decrease the incidence of PND. The purpose of this study was to evaluate the effects of different cerebral function monitoring (electroencephalography (EEG) and regional cerebral oxygen saturation (rSO_2_) monitoring) on PND based on the data from randomized controlled trials (RCTs).

**Methods:**

The electronic databases of Ovid MEDLINE, PubMed, EMBASE, Cochrane Library database were systematically searched using the indicated keywords from their inception to April 2020. The odds ratio (OR) or mean difference (MD) with 95% confidence interval (CI) were employed to analyze the data. Heterogeneity across analyzed studies was assessed with chi-square test and I^2^ test.

**Results:**

Twenty two RCTs with 6356 patients were included in the final analysis. Data from 12 studies including 4976 patients were analyzed to assess the association between the EEG-guided anesthesia and PND. The results showed that EEG-guided anesthesia could reduce the incidence of POD in patients undergoing non-cardiac surgery (OR: 0.73; 95% CI: 0.57–0.95; *P* = 0.02), but had no effect on patients undergoing cardiac surgery (OR: 0.44; 95% CI: 0.05–3.54; *P* = 0.44). The use of intraoperative EEG monitoring reduced the incidence of POCD up to 3 months after the surgery (OR: 0.69; 95% CI: 0.49–0.96; *P* = 0.03), but the incidence of early POCD remained unaffected (OR: 0.61; 95% CI: 0.35–1.07; *P* = 0.09). The remaining 10 studies compared the effect of rSO_2_ monitoring to routine care in a total of 1380 participants on the incidence of PND. The results indicated that intraoperative monitoring of rSO_2_ could reduce the incidence of POCD (OR 0.53, 95% CI 0.39–0.73; *P* < 0.0001), whereas no significant difference was found regarding the incidence of POD (OR: 0.74; 95% CI: 0.48–1.14; *P* = 0.17).

**Conclusions:**

The findings in the present study indicated that intraoperative use of EEG or/and rSO_2_ monitor could decrease the risk of PND.

**Trial registration:**

PROSPREO registration number: CRD42019130512.

## Background

Protecting brain functions is one of the essences of anesthesia practice. There is an increasing concern about the potential effects of anesthetics on perioperative neurocognitive disorders (PND) [1]. PND is a common complication after major surgeries including postoperative delirium (POD), postoperative cognitive decline (POCD) and delayed neurocognitive recovery. Its incidence rate ranges from 10 to 50% in general population. In high-risk patients, the incidence rate could reach as high as 50–70% [2–4]. PND is associated with several poor prognosis, such as higher mortality, long-term cognitive decline, dementia, re-admission and prolonged length of hospitalization. It also increases the financial burdens to the public, reaching up to $ 16 billion for US health care cost every year [5–10].

It has been revealed by several studies that the risk of PND is increased by either that excessively deep anesthesia or lower level of regional cerebral oxygen saturation during the operation [11–13]. These findings provided evidence to support the necessities of maintaining proper depth of anesthesia and enhancing cerebral perfusion, therefore increasing the level of cerebral oxygen saturation. Various monitoring technologies have become available to monitor the cerebral function. For example, electroencephalography (EEG) is a commonly used method to monitor the depth of anesthesia [14]. Regional cerebral oxygen saturation (rSO_2_) monitoring detected by near infrared reflected spectroscopy (NIRS) can be used to monitor cerebral saturation and to alert cerebral ischemia [15]. Previous meta-analysis indicated that the use of cerebral monitors during surgery correlated with a reduced risk of PND [16, 17]. However, this conclusion appeared to be controversial as some recently published large randomized controlled trials showed that the use of cerebral monitors didn’t benefit the reduction of PND incidence after major surgeries [18, 19].

To better understand the effects of cerebral function monitoring on PND and to provide clearer guidance to clinicians, we conducted this systematic review to investigate the relationship between intraoperative cerebral function monitoring and the adverse clinical outcomes.

## Methods

This review was conducted and reported following the Preferred Reporting Items for Systematic Reviews and Meta-Analysis Statement (PRISMA) guidelines [20]. This systematic review and meta-analysis had been registered in the international prospective register of systematic reviews (CRD42019130512 https://www.crd.york.ac.uk/prospero/display_record.php?RecordID=130512).

### Search strategy

Two investigators (DL and DXC) performed a systematic search in the databases of Ovid MEDLINE, PubMed, EMBASE, Cochrane Library database, and other databases updated to April 2020. The searching keywords included “cerebral monitoring”, “electroencephalography”, “cerebral oxygenation”, “postoperative delirium”, “postoperative cognitive decline”, and “randomized controlled trial”. The search terms were modified for each database. Any conflict about search results between the two investigators (DL and DXC) was resolved by discussion and the consensus was reached. The literature search strategy is provided in Additional file 1: Material 1.

### Eligibility criteria

Prior to the systematic review and meta-analysis, the inclusion criteria were predetermined by all authors. Inclusion criteria were as the following: (1) the study was randomized controlled trial (RCT), regardless of the language and status; (2) included patients were adults aged 18 years or older who underwent general anesthesia for surgery; (3) the incidence of PND under the EEG or rSO_2_ monitoring was compared to the PND outcome without the usage of EEG or rSO_2_ monitoring in the study; (4) the occurrence of PND evaluated by validated scale was reported in the study. The exclusion criteria were: (1) non-randomized studies; (2) non full-text studies; (3) ongoing studies; (4) the outcome data could not be extracted and used to analyze.

### Data collection and quality assessment

Data was extracted by two investigators (DL and DXC) independently using a standardized form based on the Population Interventions Comparisons Outcomes (PICO) approach. The extracted information included the first author, year of publication, study design, sample size, outcome variables and assessment scale, summative results and conclusion. The methodological quality of the included studies was with using the Cochrane risk of bias scale, which contains seven specified domains [21]. Risk of bias were classified as high, low or unclear for each item. The methodological quality assessment was conducted by two investigators independently, and the occurred conflicts were resolved by a third investigator (QL) referring to the original article, if any.

### Statistical analysis

Data analyses were performed using the Review Manager (version 5.3) software. The inspection level for the pooled data were two-sided, and *P* < 0.05 was regarded as statistically significant. The odds ratio (OR) and mean difference (MD) with 95% confidence interval (CI) were employed to analyze the categories and continuous data. Heterogeneity across studies was assessed with chi-square test and I^2^ test, and I^2^ > 50% or *P* < 0.10 was considered as significantly heterogenous. The random-effect model was adopted if the heterogeneity existed among the studies, whereas the fixed-effect model was applied if no significant heterogeneity was detected. Sensitivity analysis was conducted to assess the impact of single study to the overall analysis [22–25]. Publication bias was assessed by using the funnel plot test.

## Results

### Literature search

**Fig. 1:**
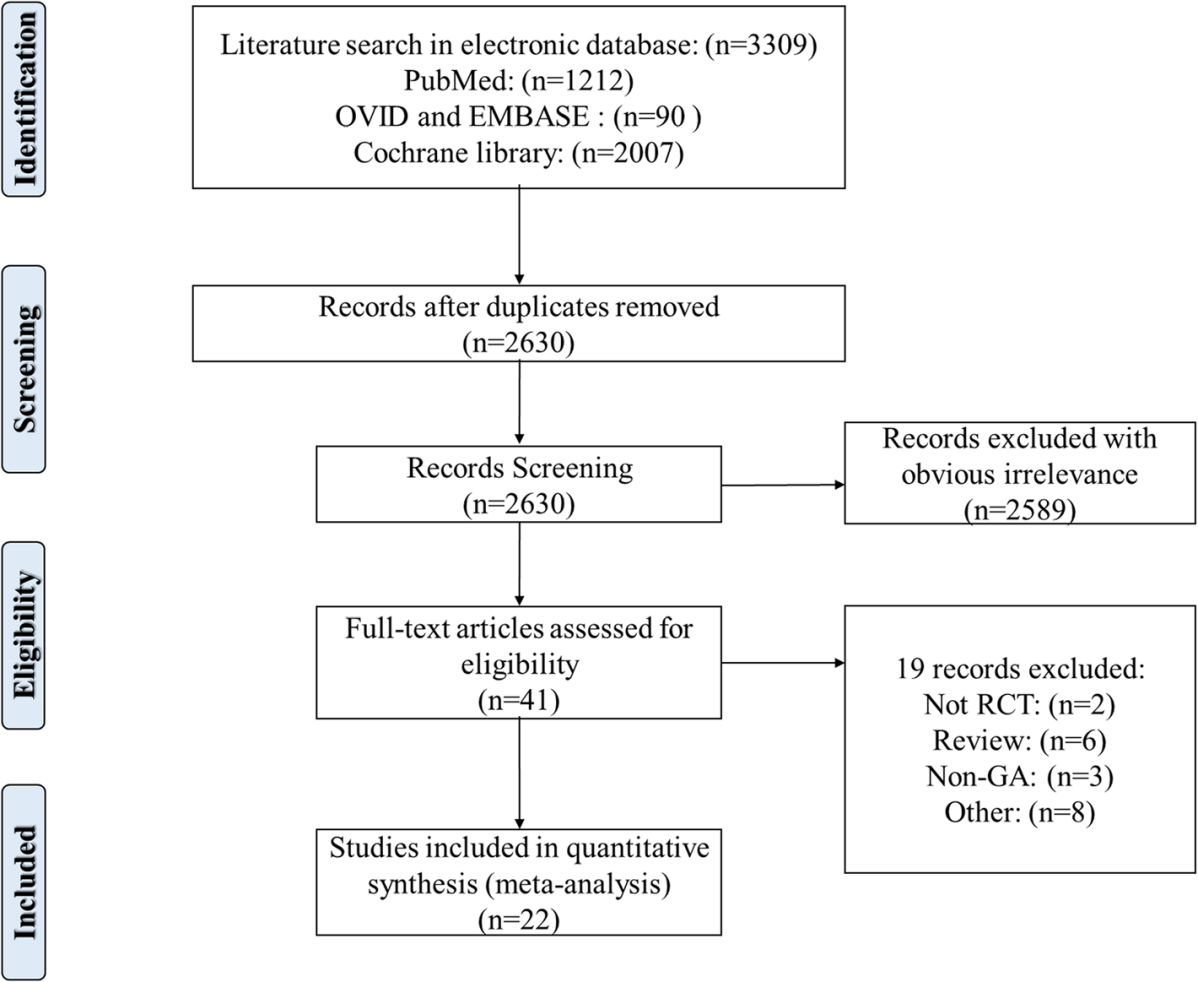
Flow diagram of the literature search and trials screening process

The initial search in PubMed, Ovid, EMBASE, Cochrane library, and other databases identified 3309 reports. Duplicates removal reduced the number of reports to 2630. Then, 2589 studies were further excluded after reviewing the title and abstracts. The full text of the remaining 41 studies were retrieved for evaluation, 19 out of the 41 studies were further excluded due to one or more of the following reasons: not RCT (*n* = 2); review (*n* = 6); non-general anesthesia patients (*n* = 3); or other studies which data could not be extracted or used to analyze (*n* = 8). Reviewing the reference lists of the retrieved studies did not identify any new eligible study. Finally, 22 RCTs were included in the present this review [18, 19, 26–45]. A flow diagram illustrating the literature search and trials screening process was shown in Fig. 1.

### Characteristics of included studies

**Fig. 2:**
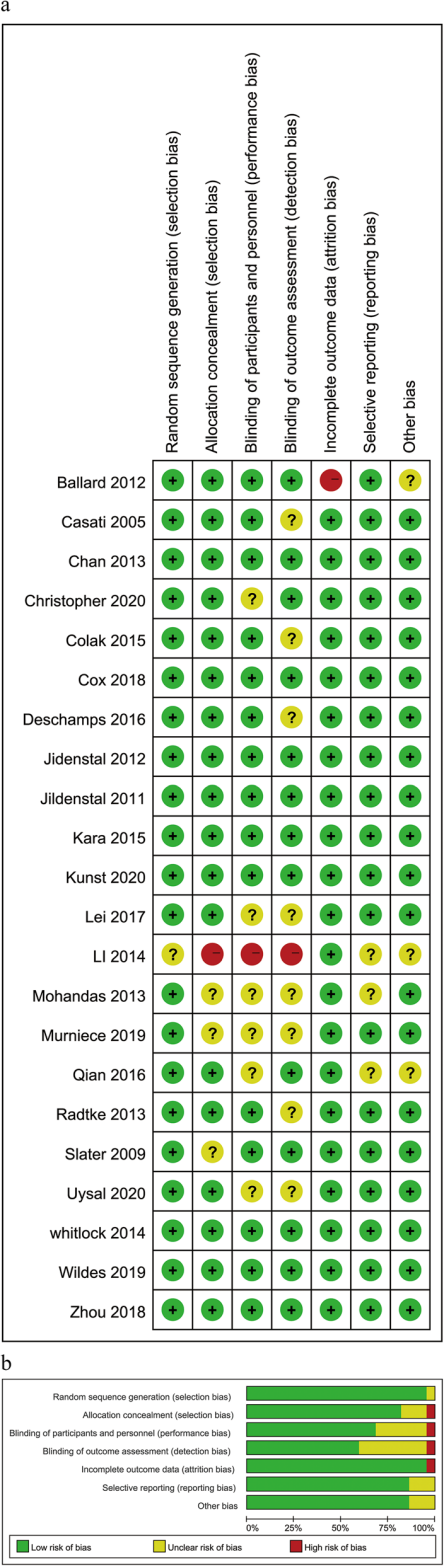
The risk of bias assessment of included studies. (**a**, risk of bias summary: review authors’ judgements of each risk of bias item for each included study; **b**, risk of bias graph: review authors’ judgements about each risk of bias item presented as percentages across all included studies)

As listed in Table 1, a total number of 6356 patients included in the 22 RCTs were enrolled in this meta-analysis [18, 19, 26–45]. Among these 22 RCTs, 10 studies used EEG as the guide for anesthesia depth [18, 26–34], 10 studies evaluated the effects of rSO_2_ monitoring on PND [19, 35–43] and the last 2 trials deployed both of the two monitoring [44, 45]. Ten out of the 22 trials included patients who underwent cardiac surgery [18, 19, 27, 36, 38–41, 43, 45], whereas the other 12 studies were conducted in among patients undergoing non-cardiac major surgery, including abdominal surgery, ENT surgery, hip fracture repair surgeries and others [26, 28–34, 37, 39, 42, 44]. The risk of bias of included studies was assessed and the result was shown in Fig. 2. Two studies were at the high level in terms of risk of bias. One of them was lacking of the methods of allocation and blinding [33], and the other had a high dropout rate [44]. Eleven studies [19, 30, 32, 34–36, 38, 40–43] were at unclear risk of bias due to the unclear blinding of outcome assessments (detection bias) or unclear blinding of participants and study personnel (performance bias). The remaining 9 studies rated as low in terms of the risk of bias.

**Table 1:** Characteristics of included studies

Study	Population	The type of surgery	Intervention	Major outcome	Assessment scales	Conclusion
**EEG**						
**Wildes 2019** [18]	60 years or older	Major surgery (cardiac = 459; non-cardiac =754)	N = 1213 BIS-guided = 604 Routine care = 609	POD (postoperative day1 -day5)	CAM/CAM-ICU	There was no difference between two groups.
**Christopher 2020** [34]	65 years or older	Major Noncardiac Surgery	N = 204 PSI- guided =102 Routine care =102	POD (postoperative day 1–3) EEG suppression ratio	CAM	The incidence of delirium was not found to be different between two groups.
**Radtke 2013** [30]	60 years or older	Non-cardiac surgery	*N* = 1155 BIS-guided = 575 Routine care = 580	POD (postoperative day 1–7, twice a day) POCD (postoperative 7 days and 3 months)	DSM IV CANTAB	The routine care group had a higher incidence of POD compared with the BIS-guided group.
**Zhou 2018** [31]	65–75 years old	Resection of colon carcinoma	*N* = 81 BIS-guided = 41 Routine care = 40	POD (postoperative day 1 -day5)	CAM	The incidence of POD was significantly lower in the BIS-guided group compared with the routine care group.
**Qian 2016** [32]	65–85 years old	Gastrointestinal surgery	*N* = 180 BIS-guided = 90 Routine care = 90	POD (postoperative day 1 -day7)	CAM	General anesthesia under BIS monitoring can reduce the incidence and duration of POD
**Li 2014** [33]	65–83 years old	Upper abdominal operation	*N* = 295 BIS-guided = 147 Routine care = 148	POD (postoperative day 1 -day3)	DSM IV	The use of BIS guidance reduced the incidence of postoperative delirium
**Whitlock 2014** [27]	18 years or older	Cardiac or thoracic surgery	*N* = 310 BIS-guided = 149 ETAC-guided = 161	POD (twice daily until postoperative day 10 or ICU discharge)	CAM-ICU	There was no difference between two groups.
**Jidenstal 2012** [28]	40–94 years old	ENT surgery	*N* = 32 AEP-guided = 16 Routine care = 16	POD (postoperative day1) POCD (postoperative day 1 and 1 month)	CAM	AEP-guided anesthesia decreased the risk of early POCD rather than early POD.
**Chan 2013** [29]	60 years or older	Major non-cardiac surgery	*N* = 902 BIS-guided = 450 Routine care = 452	POD (in-hospital) POCD (postoperative 1 week and 3 months)	CAM	BIS-guided anesthesia reduced the risk of postoperative delirium.
**Jidenstal 2011** [26]	18–92 years old	Ophthalmic surgery	*N* = 450 AEP-guided = 224 Routine care = 226	POCD (postoperative 1 day, 1 week or 1 month)	MMT and CFQ)	Patients with AEP-guided anaesthesia had a lower risk of early postoperative cognitive decline.
**Kunst 2020** [45]	64 years or older	CABG	*N* = 82 BIS and rSO_2_- guided =42 Routine care =40	POD (postoperative day 3–5) POCD (postoperative day 3–5, 6 weeks)	CAM MMSE	Optimizing both depth of anesthesia and rSO2 in elderly patients undergoing cardiac surgery resulted in a significant reduction in the postoperative delirium.
**Ballard 2012** [44]	70 years or older	Abdominal and Orthopaedic surgery	*N* = 72 BIS and rSO_2_- guided =34 Routine care = 38	POCD (postoperative 1 week, 12 weeks, 52 weeks)	MMSE	Intraoperative monitoring of anaesthetic depth and cerebral oxygenation can reduce post-operative cognitive impairment.
**Cerebral Oxygenation Monitoring**						
**Casati 2005** [35]	65 years or older	Major abdominal surgery	*N* = 122 rSO_2_- guided =56 Routine care = 66	POCD (postoperative 1 week)	MMSE	Using rSO_2_ monitoring seems to result in less cognitive decline.
**Colak 2015** [36]	40–80 years old	CABG	*N* = 190 rSO_2_- guided =94 Routine care = 96	POCD (postoperative 1 week) POD (postoperative 1 week)	CTT and GP test	The use of INVOS monitoring has a predictive value in terms of lower incidence of early postoperative cognitive decline.
**Kara 2015** [39]	–	CABG	*N* = 79 rSO_2_- guided =43 Routine care = 36	POCD	MoCA	Intraoperative NIRS usage can decrease the incidence of POCD
**Mohandas 2013** [40]	–	Open heart surgery	*N* = 100 rSO_2_- guided =50 Routine care = 50	POCD (postoperative 1 week and 3 months)	MMSE, ASEM	Intraoperative monitoring of rSO_2_ can significantly decrease the incidence of postoperative neurocognitive decline.
**Murniece 2019** [42]	18 years or older	Spinal Neurosurgery	*N* = 34 rSO_2_- guided =23 Routine care = 11	POCD (postoperative 1 week and 3 months)	MoCA	Use of the NIRS-based clinical algorithm can help to avoid POCD in patients.
**Slater 2009** [43]	–	CABG	*N* = 240 rSO_2_-guided = 125 Routine care = 115	POCD (postoperative 1 week and 3 months)	MMSE, ASEM	There was no difference between two groups on POCD.
**Deschamps 2016** [38]	18 years or older	High-risk Cardiac Surgery	*N* = 201 rSO_2_- guided =102 Routine care = 99	POD (postoperative 3 months)	DSM IV	There was no difference between two groups on POD.
**Lei 2017** [41]	60 years or older	Cardiac surgery	*N* = 249 rSO_2_- guided =123 Routine care = 126	POD (postoperative 12 h-7 days)	CAM/CAM-ICU	Three was no difference in the incidence of POD between the intervention group and control group.
**Uysal 2020** [19]	18 years or older	Cardiac surgery	*N* = 125 rSO_2_- guided =59 Routine care = 66	POD-ICU (postoperative 24 h, 3 and 6 months	Cognitive Stability Index HeadMinder	Three was no difference in the incidence of POD between the intervention group and control group.
**Cox 2018** [37]	18 and 85 years	Arthroscopic shoulder surgery	*N* = 40 rSO_2_- guided =20 Routine care = 20	POCD (postoperatively, before discharge; postoperative 2 weeks and 6 weeks)	MoCA	No difference between two groups.

*Abbreviations*: *EEG* Electroencephalography, *POD* Postoperative delirium, *BIS* Bispectral index, *AEP* Auditory evoked potential, *rSO*_*2*_ Regional cerebral oxygen saturations, *POCD* Postoperative cognitive decline, *ETAC* End-tidal anesthetic concentration, *ENT* Ear, nose, and throat, *CABG* Coronary artery bypass graft surgery, *CAM* Confusion assessment method, *DSM IV* Diagnostic and Statistical Manual of Mental Disorders, *MMSE* Mini mental state examination, *MoCA* Montreal mognitive assessment, *ASEM* Antisaccadic eye movement test, *CTT* Color Trail Test, *GP test* Grooved-Pegboard Test, *MMT* The mini-mental test, *CFQ* Cognitive Failure Questionnaire

### Cerebral functional monitoring and perioperative neurocognitive disorders (PND)

#### Electroencephalography (EEG) guided anesthesia

##### Postoperative delirium (POD)

**Fig. 3:**
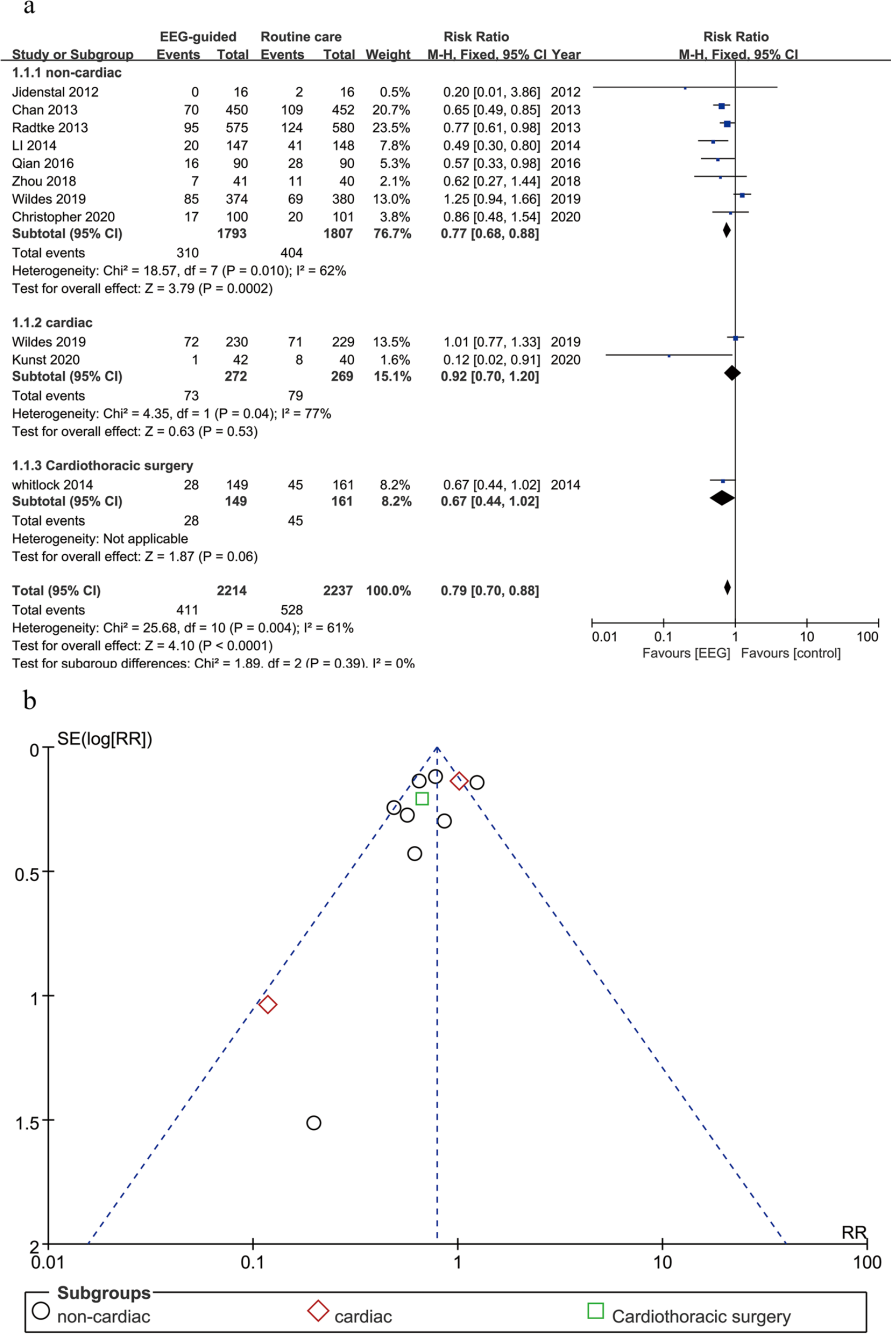
Postoperative delirium (POD) of EEG guided arm vs routine care arm. (**a**, forest plot of POD; **b**, funnel plot of POD)

After pooling and analyzing the data from the 10 studies using EEG to guide the depth of anesthesia (*n* = 4451, EEG monitoring = 2214, routine care = 2237), it is noticed that in general, the EEG-guided anesthesia group had a reduced risk of POD compared to the group of routine care (OR: 0.75; 95% CI: 0.60–0.93; *P* = 0.008) (Fig. 3a) with a significant, heterogeneity detected among the included studies (*P* = 0.004, I^2^ = 61%). Then we divided the participants into non-cardiac or cardiac subgroups according to the types of surgeries that patients received and re-analyzed the effect of EEG on the risk of POD. The results demonstrated that in the non-cardiac surgery subgroup (8 studies, *n* = 3600, EEG monitoring = 1793, non-EEG group = 1807), the use of EEG-guided anesthesia correlated with a reduction of POD incidence (OR: 0.73; 95% CI: 0.57–0.95; *P* = 0.02), whereas in the cardiac surgery subgroup, no correlation was detected between the use of EEG and the reduction of POD incidence (2 studies, *n* = 541, EEG monitoring = 272, non-EEG group = 269; OR: 0.44; 95% CI: 0.05–3.54; *P* = 0.44). It is noted that the study conducted by Whitlock et al. was excluded from the subgroup analysis because it included both cardiac surgery and thoracic surgery without detailed information on the number of patients in the each subgroup. The funnel plot demonstrated that no publication bias existed among the included studies (Fig. 3b).

##### Postoperative cognitive decline (POCD)

**Fig. 4:**
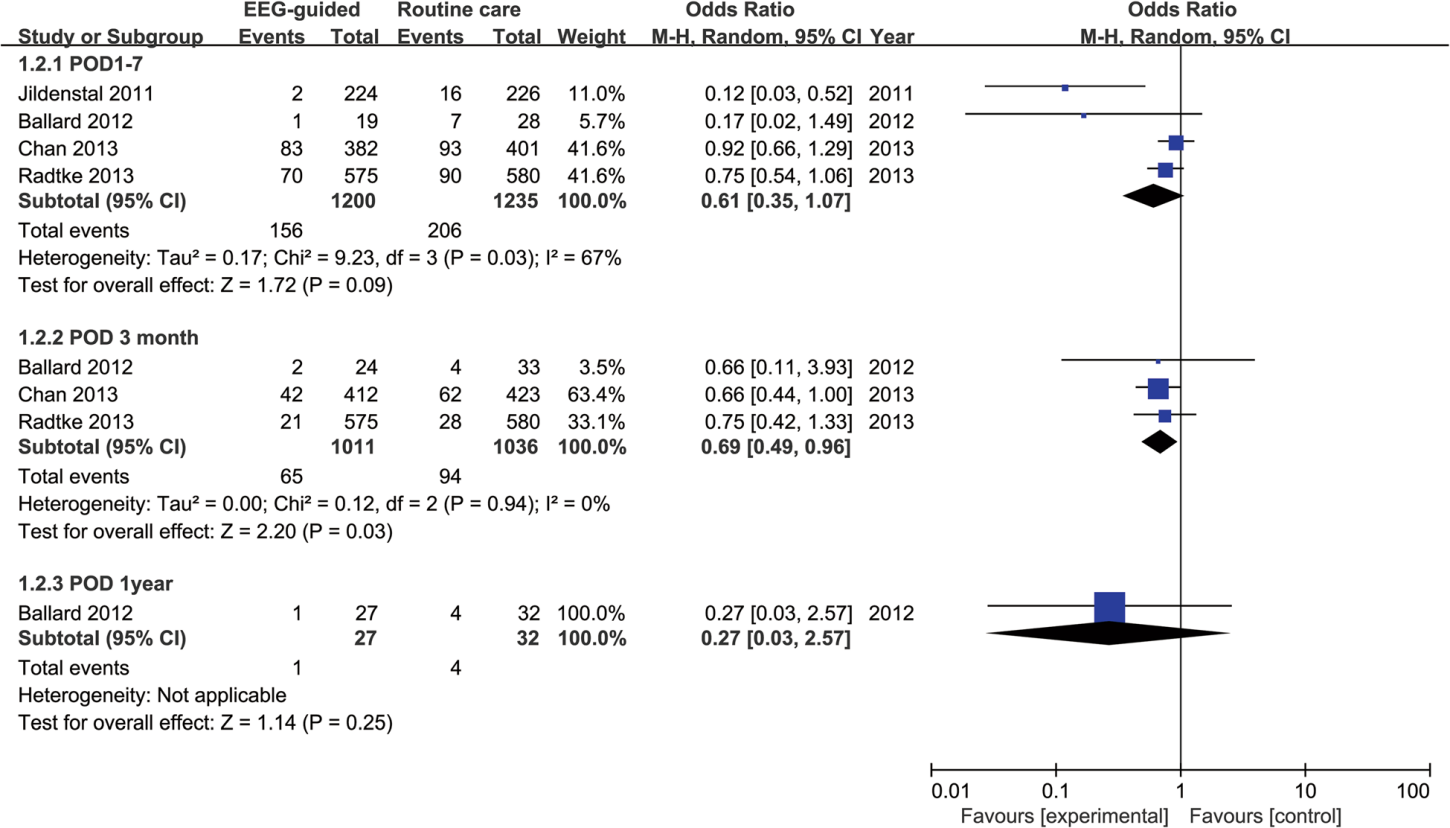
Postoperative cognitive decline (POCD) of EEG guided arm vs routine care arm

Four studies (*n* = 2435, EEG monitoring = 1200, routine care = 1235) reported the incidence of POCD within 1–7 days after surgery after non-cardiac surgery, and three of them also reported the incidence of POCD 3 months after the surgery (*n* = 2047, EEG monitoring = 1011, routine care = 1036). It was found that EEG-guided anesthesia did not reduce the incidence of POCD in the early postoperative stage (OR: 0.61; 95% CI: 0.35–1.07; *P* = 0.09). However, the incidence of POCD 3 months after the surgery was reduced upon the use of intraoperative EEG monitoring (OR: 0.69; 95% CI: 0.49–0.96; *P* = 0.03). Only one study by Ballard and his colleagues, reported the incidence of POCD at the time of 1 year postoperatively (*n* = 59, EEG monitoring = 27, routine care = 32), but it did not suggest the advantages of EEG monitoring with respect to the incidence of POCD (OR: 0.27; 95% CI: 0.03–2.57; *P* = 0.25) (Fig. 4).

#### Regional cerebral oxygen saturation (rSO_2_) monitoring

##### Postoperative delirium (POD)

**Fig. 5:**
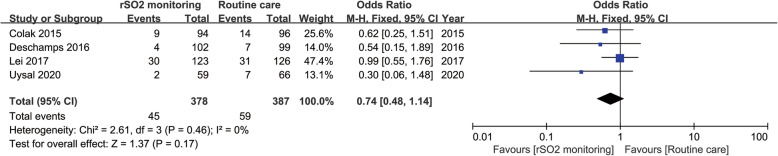
Postoperative delirium (POD) of rSO_2_ Monitoring arm vs routine care arm

Four studies including 765 patients undergoing cardiac surgery reported the outcome of incidence of POD between the rSO_2_ monitoring group (*n* = 378) and the routine care group (*n* = 387). All the four studies showed no difference in terms of POD between the two groups. Our meta-analysis also revealed a comparable result about the incidence of POD between the cerebral oxygenation monitoring group and the routine care group (OR: 0.74; 95% CI: 0.48–1.14; *P* = 0.17) (Fig. 5).

##### Postoperative cognitive decline (POCD)

**Fig. 6:**
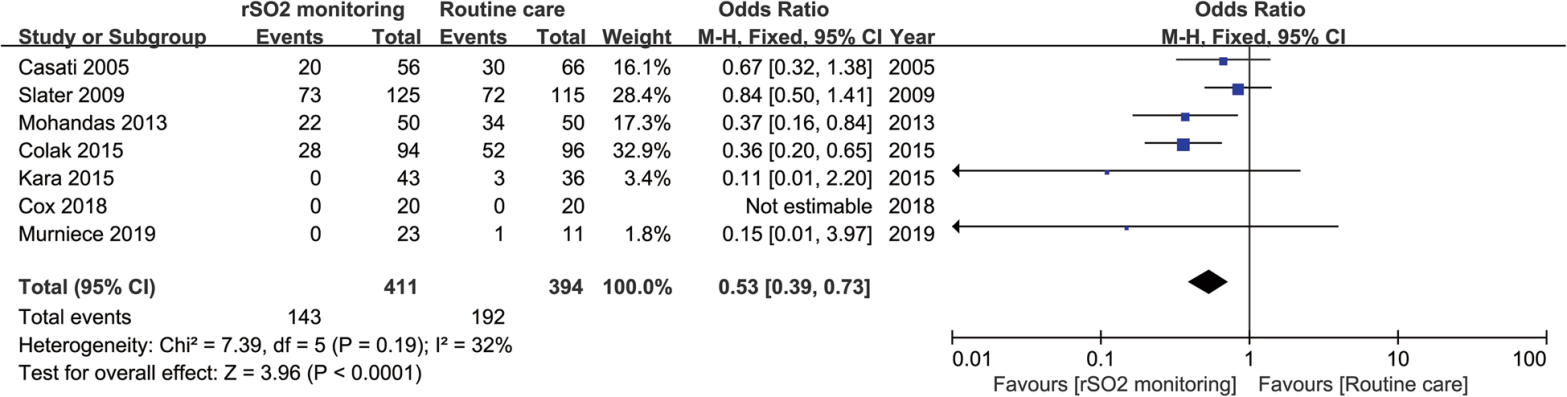
Postoperative cognitive decline (POCD) of rSO_2_ arm vs routine care arm

Seven studies involving 805 patients analyzed the effect of rSO_2_ monitoring on the incidence of POCD after major surgeries compared to that of routine care (*n* = 805, rSO_2_ monitoring group, *n* = 411; routine care, *n* = 394). Among these 7 studies, Slater et al. focused on cardiac surgery [43], while Cox and his colleagues reported the result of their work in non-cardiac surgery [37]. Despite the different types of surgeries studied, both groups reported no difference found between the rSO_2_ monitoring group and the routine care group in terms of the incidence of POCD. However, the remaining five trials, three of which conducted in patients undertaking cardiac surgeries [36, 39, 40] and two of which performed in patients undertaking non-cardiac surgeries [35, 42], reported that the incidence of POCD in the routine care group was significantly higher than that of rSO_2_ monitoring group. Our meta-analysis also revealed a significant lower incidence of the POCD in the rSO_2_ monitoring group (OR: 0.53, 95% CI 0.39–0.73; *P* < 0.0001) (Fig. 6) compared to that of routine care group without heterogeneity detected (*P* = 0.19; I^2^ = 32%).

## Discussion

In the present systematic review and meta-analysis, data of 6356 patients from 22 RCTs was analyzed, including 3169 patients who received EEG or/and rSO_2_ monitoring, and 3187 patients who received routine care. In non-cardiac surgery patients, we found the incidence of POD significantly decreased in EEG-guided anesthesia group compared to that of routine care group. Both EEG-guided anesthesia and rSO_2_ monitoring were correlated with a significant lower incidence of POCD despite the types of surgery.

### EEG-guided anesthesia

Twenty studies including 4976 patients assessed the association between the EEG-guided anesthesia and PND [18, 26–34]. Our meta-analysis showed that EEG-guided anesthesia could reduce the incidence of POD in patients undergoing non-cardiac surgeries but not cardiac surgery patients. In addition, deployment of intraoperative EEG monitoring could reduce the incidence of POCD up to 3 months after the surgery, but had no effect on the incidence of early POCD.

Prior to our study, Punjasawadwong et al. [46] and Kristen et al. [16] have performed two meta-analyses separately, each included 3 RCTs (*n* = 2197) and 5 RCTs (*n* = 2654) respectively, to evaluate the impact of EEG monitoring on POD and POCD. These two meta-analyses both reported that the EEG-guided anesthesia could reduce the incidence of POD. However, Kristen et al. did not draw a conclusion on whether EEG monitoring affect POCD due to the high heterogeneity among their included RCTs [16]. Both studies pointed out that the quality of the research evidence was moderate, and further studies should be required to clarify whether the appropriate cerebral function monitoring during surgery can reduce the incidence of PND. Recently, several studies have further explore this important issue. It is worth noting that a large-sample RCT (*n* = 1213) conducted by Wildes and his colleagues proposed that EEG-guided anesthesia cannot reduce the incidence of POD [18], which is inconsistent with the results from previous large-sample studies [29, 30]. In 2013, Chan et al. performed an RCT including 902 patients and revealed that the incidence rate of PND was lower in patients receiving EEG-guided anesthesia than that in patients receiving routine care [29]. In addition, Radtke and his colleagues analyzed data extracted from 1155 patients and concluded that EEG monitoring correlated with a significant reduction of POD incidence and a decreasing tendency on incidence of POCD [30]. The discrepant findings among the above-mentioned studies may attribute to the differences of their methodology and the heterogeneity of the studied population. Compared to the studies by Chan et al. or Radtke et al. [29, 30], the study conducted by Wildes et al. [18] included patients with more severe conditions as more than 30% of the patients in Wildes’ study had ASA ≥ 3, or had a history of falls, or planned cardiothoracic surgery, which might partially eliminate the effect of EEG on POD [47–49]. In our study, a similar conclusion that using EEG cannot reduce the incidence of POD was drawn in the subgroup of patients who underwent cardiac surgeries. For these high-risk patients, it is recommended by several clinical practice guidelines that a multi-component strategy is required to prevent the incidence of POD, indicating that a single approach of monitoring has a limited role in preventing the high-risk patients from POD [50, 51]. In our study, heterogeneity among included studies existed throughout the analysis except the subgroup analysis. Further large-scale RCTs should be conducted to confirm the conclusion.

The underlying mechanisms of the POD prevention by EEG monitoring remains unclear. One hypothesis is that the use of EEG monitoring makes it possible to avoid excessively anesthesia, therefore to specifically reduce the incidence and cumulative duration of intraoperative burst suppression. Previous studies have shown that burst suppression is an independent risk factor of POD [52, 53]. In addition, Hesse et al. have demonstrated that every incidence of burst suppression during the anesthesia maintenance was associated with a 75% increase in odds of POD [54]. Furthermore, higher incidence or longer duration of burst suppression are significantly associated with the incidence of POD [55, 56]. On the other hand, the use of EEG monitoring also reduced the dosage of general anesthetics, such as volatile agents and propofol [57, 58]. Previous studies have reported that excessively exposure to potent volatile agents might increase the incidence of POD [59]. Particularly, most of these studies were performed in geriatric patients whose aging brains were more sensitive to anesthetic agents, therefore, were more likely to experience the burst suppression and POD [60, 61].

### Regional cerebral oxygenation monitoring

1380 participants from 10 studies comparing the effect of rSO_2_ monitoring to routine care was included in our study [19, 35–43]. The results indicated that intraoperative monitoring of cerebral oxygenation could reduce POCD, but have no effect on POD.

Prior to our research, Yu et al. conducted a meta-analysis to evaluate the impact of cerebral near infrared reflected spectroscopy (NIRS) monitoring on the following clinical outcomes, including cerebral oxygen desaturation events, neurological outcomes, non-neurological outcomes and socioeconomic impact. The results from the study suggested that the effects of rSO_2_ monitoring on POCD or POD are uncertain due to the low quality of the evidence and high heterogeneity among included studies [17].

Since the total amount of oxygen consumed by the brain is about 20% of body oxygen supply, the cerebral function is extremely vulnerable to hypoxemia. A study found that 50 to 75% of patients undergoing cardiac surgery experienced once or more rSO_2_ desaturations during cardiopulmonary bypass (CPB) [62], and that prolonged low rSO_2_ values have been associated with significantly higher risks of POCD [63, 64]. Evidence indicates that perioperative rSO_2_ levels below a certain level (50–60%) are associated with an increase in neurological complications and an increase in mortality. It also suggested not to interpret rSO_2_ levels based on absolute values rather than follow the trend analysis instead, by interpreting the relative changes of rSO_2_ levels with respect to an individual baseline value [65]. Therefore, rSO_2_ monitoring has been used to mitigate the cerebral oxygen desaturation during surgery.

NIRS is an emerging noninvasive technique of monitoring brain oxygenation and increasingly being used in various clinical settings. This provides an opportunity for early recognition of imbalances of oxygen delivery and consumption and rSO_2_ desaturation [66]. Clinicians can take more active treatment measures to prevent prolonged rSO_2_ desaturation, thereby avoiding neurological and other major complications. However, the clinical benefits of this technology have been questioned. In a multicenter prospective randomized study conducted by Deschamps et al. which included 201 patients [38], the authors found that NIRS-guided intervention can prevented the decreases of rSO_2_ in cardiac surgery but did not reduce the incidence of PND. These findings were consistent with two single-center RCTs conducted by Lei et al. and Slater et al. respectively [41, 43]. Although our meta-analysis suggest that rSO_2_ monitoring can reduce POCD, the quality of the included studies is not uniform, and the definition and evaluation methods of POCD were also different. Therefore, further and larger multi-center RCTs are needed to confirm our conclusions.

In our meta-analysis, there were two studies evaluated the effects of the combination of combined use of EEG monitoring and regional cerebral saturation monitoring on reducing PND [44, 45]. The RCT conducted by Ballard and colleagues showed that the use of EEG and rSO_2_ monitor can significantly reduce the incidence of POCD at1, 12 and 52 weeks postoperatively [44]. But another study conducted by Kunst et al. found that in elderly patients undergoing coronary artery bypass graft surgery (CABG), the combined use of EEG and rSO_2_ monitoring can reduce the incidence of POD rather than POCD [45]. Nevertheless, It was reported in both studies suggested that the combined use of EEG and rSO_2_ monitoring reduced the incidence of PND. This result suggested that combination of multiple monitoring approaches is better than one.

NIRS guided rSO_2_ monitoring can provide clinicians with information on the quality of cerebral oxygen delivery. However, NIRS cannot reflect brain function. EEG can provided objective measures assessing brain cortical function according to differently dynamic waveform, and the generation of electrical activity requires adequate cerebral perfusion and cerebral oxygen. Previous studies showed the observed EEG pattern consisted only of voltage suppression at rSO_2_ level was less than 20%, while the delta background activity was seen at rSO_2_ level was greater than 40%. The emergence of delta background activity may be a sign of cortical functional recovery [67]. In addition, Horst et al. investigated the relationship between rSO_2_ and EEG in preterm infants. The authors concluded that there is a significant relationship between electrocortical activity and oxygen consumption as cerebral oxygen metabolism increases with increasing EEG amplitude [68]. So by the combination of EEG data and NIRS data, the clinicians were able to potentially assess the impact of cerebral oxygen delivery on cortical function as determined via EEG patterns [67]. Both the increased brain oxygen consumption and the decreased brain electrical activity reflect the compromised cerebral oxygen delivery or perfusion, and early warning and intervention may prevent the neurological impairment. Although the potential mechanism of rSO_2_ on EEG under anesthesia has not been clarified, considering the possibility of intraoperative burst suppression caused by excessive anesthesia and cerebral oxygen desaturation caused by insufficient perfusion, the collaborative application of both monitoring may provide benefits for reducing the incidence of PND.

Several limitations of this meta-analysis should be acknowledged: (1) smaller number of included trials and less solidarity of the results due to the absence of adjustment variables such as age, gender and the type of surgery; (2) there were different scales to assess the incidence of POD or POCD in our included studies, such as CAM/CAM-ICU, DSM-IV, MMSE and other scales which had different specificity and sensitivity. However, we carefully read the definitions of POD and POCD in each original text to extract the data with as little heterogeneity as possible; (3) this study is based on the published articles, the publication bias is inevitable; (4) the analysis of this study is based on data at study-level, whereas the original data from individual patients was not available.

## Conclusions

In conclusion, the findings of our study indicated that the use of EEG or/and rSO_2_ monitor correlated with a lower risk of PND. Based on this, we recommend intraoperative EEG or/and rSO_2_ monitoring during surgery to decrease the risk of PND. In addition, for high-risk patients, multiple monitoring approaches should be combined to optimize the anesthesia management and to prevent the incidence of PND. Further research should be conducted to verify the identified correlation between the use of EEG or/and rSO_2_ monitoring and PND.

## Supplementary information

**Additional file 1:** Literature search strategy.

**Additional file 2:** PRISMA Checklist.

## Data Availability

The datasets used and/or analysed during the current study are available from the corresponding author on reasonable request.

## Abbreviations

PND: Perioperative Neurocognitive Disorders
EEG: Electroencephalography
POD: Postoperative delirium
rSO_2_: Regional cerebral oxygen saturations
POCD: Postoperative cognitive decline
NIRS: Near infrared reflected spectroscopy
PICO: Population Interventions Comparisons Outcomes
ETAC: End-tidal anesthetic concentration
ENT: Ear, nose, and throat
CABG: Coronary artery bypass graft surgery
CAM: Confusion assessment method
DSM IV: Diagnostic and Statistical Manual of Mental Disorders
MMSE: Mini mental state examination
CPB: Cardiopulmonary bypass
CI: Confidence interval
OR: Odds ratio
RCTs: Randomized controlled trials
MD: Mean difference
