# Supplementary material for: Effects of electroencephalography and regional cerebral oxygen saturation monitoring on perioperative neurocognitive disorders: a systematic review and meta-analysis
Source: BMC Anesthesiol. 2020 Sep 30;20:254. doi: 10.1186/s12871-020-01163-y (PMC7526409; doi:10.1186/s12871-020-01163-y)
Supplement: Supplementary file 1 — Additional file 1: Literature search strategy. [file 12871_2020_1163_MOESM1_ESM.docx]

**Additional file 1: Material 1(Literature search strategy)**

(((("electroencephalographies"[All Fields] OR "electroencephalography"[MeSH Terms]) OR "electroencephalography"[All Fields]) OR (((((("cerebrally"[All Fields] OR "cerebrum"[MeSH Terms]) OR "cerebrum"[All Fields]) OR "cerebral"[All Fields]) OR "brain"[MeSH Terms]) OR "brain"[All Fields]) AND ((((((((((((("monitor s"[All Fields] OR "monitorable"[All Fields]) OR "monitored"[All Fields]) OR "monitoring"[All Fields]) OR "monitoring s"[All Fields]) OR "monitoring, physiologic"[MeSH Terms]) OR ("monitoring"[All Fields] AND "physiologic"[All Fields])) OR "physiologic monitoring"[All Fields]) OR "monitor"[All Fields]) OR "monitorings"[All Fields]) OR "monitorization"[All Fields]) OR "monitorize"[All Fields]) OR "monitorized"[All Fields]) OR "monitors"[All Fields]))) OR (((((("cerebrally"[All Fields] OR "cerebrum"[MeSH Terms]) OR "cerebrum"[All Fields]) OR "cerebral"[All Fields]) OR "brain"[MeSH Terms]) OR "brain"[All Fields]) AND (((((((((((((((((((("cell respiration"[MeSH Terms] OR ("cell"[All Fields] AND "respiration"[All Fields])) OR "cell respiration"[All Fields]) OR "oxygenation"[All Fields]) OR "oxygen"[MeSH Terms]) OR "oxygen"[All Fields]) OR "oxygen s"[All Fields]) OR "oxygenate"[All Fields]) OR "oxygenated"[All Fields]) OR "oxygenates"[All Fields]) OR "oxygenating"[All Fields]) OR "oxygenations"[All Fields]) OR "oxygenative"[All Fields]) OR "oxygenator s"[All Fields]) OR "oxygenators"[MeSH Terms]) OR "oxygenators"[All Fields]) OR "oxygenator"[All Fields]) OR "oxygene"[All Fields]) OR "oxygenic"[All Fields]) OR "oxygenous"[All Fields]) OR "oxygens"[All Fields]))) AND ((((((((("postoperative period"[MeSH Terms] OR ("postoperative"[All Fields] AND "period"[All Fields])) OR "postoperative period"[All Fields]) OR "postop"[All Fields]) OR "postoperative"[All Fields]) OR "postoperatively"[All Fields]) OR "postoperatives"[All Fields]) AND (("delirium"[MeSH Terms] OR "delirium"[All Fields]) OR "deliriums"[All Fields])) OR ((((((("postoperative period"[MeSH Terms] OR ("postoperative"[All Fields] AND "period"[All Fields])) OR "postoperative period"[All Fields]) OR "postop"[All Fields]) OR "postoperative"[All Fields]) OR "postoperatively"[All Fields]) OR "postoperatives"[All Fields]) AND (((("cognitive dysfunction"[MeSH Terms] OR ("cognitive"[All Fields] AND "dysfunction"[All Fields])) OR "cognitive dysfunction"[All Fields]) OR ("cognitive"[All Fields] AND "decline"[All Fields])) OR "cognitive decline"[All Fields]))) OR (("perioperative"[All Fields] OR "perioperatively"[All Fields]) AND (("neurocognitive disorders"[MeSH Terms] OR ("neurocognitive"[All Fields] AND "disorders"[All Fields])) OR "neurocognitive disorders"[All Fields])))
